# Adolescent Pregnancy and Attained Height among Black South African Girls: Matched-Pair Prospective Study

**DOI:** 10.1371/journal.pone.0147861

**Published:** 2016-01-25

**Authors:** Elizabeth A. Lundeen, Shane A. Norris, Reynaldo Martorell, Parminder S. Suchdev, Neil K. Mehta, Linda M. Richter, Aryeh D. Stein

**Affiliations:** 1 Nutrition and Health Sciences Program, Division of Biological and Biomedical Sciences, Laney Graduate School, Emory University, Atlanta, Georgia, United States of America; 2 MRC Developmental Pathways for Health Research Unit, University of the Witwatersrand, Johannesburg, South Africa; 3 Hubert Department of Global Health, Rollins School of Public Health, Emory University, Atlanta, Georgia, United States of America; 4 DST-NRF Centre of Excellence of Human Development, University of the Witwatersrand, Johannesburg, South Africa; Institute of Preventive Medicine, DENMARK

## Abstract

**Importance:**

The impact of adolescent pregnancy on offspring birth outcomes has been widely studied, but less is known about its impact on the growth of the young mother herself.

**Objective:**

To determine the association between adolescent pregnancy and attained height.

**Design:**

Prospective birth cohort study.

**Setting:**

Cohort members followed from birth to age 20 y in Soweto, South Africa.

**Participant:**

From among 840 Black females with sufficient data, we identified 54 matched pairs, in which a girl who became pregnant before the age of 17 years was matched with a girl who did not have a pregnancy by age 20 y. Pairs were matched on age at menarche and height-for-age z scores in the year before the case became pregnant (mean 15.0 y).

**Main Outcome Measures:**

The two groups were compared with respect to attained height, measured at mean age 18.5 y.

**Results:**

Mean age at conception was 15.9 years (range: 13.7 to 16.9 y). Mean height at matching was 159.4 cm in the adolescent pregnancy group and 159.3 cm in the comparison group (p = 0.3). Mean attained height was 160.4 cm in the adolescent pregnancy group and 160.3 cm in the comparison group (p = 0.7).

**Conclusions:**

Among Black females in Soweto, South Africa, adolescent pregnancy was not associated with attained height.

## Introduction

About 16 million women 15–19 years old give birth each year, with ninety-five percent of these births occurring in low- and middle-income countries.[1] The proportion of births that occur to women 15–19 y is 18% in Latin America and the Caribbean and more than 50% in sub-Saharan Africa.1 In South Africa, 27% of girls have had at least one birth by age 19.[2] The impact of adolescent pregnancy on offspring birth outcomes has been widely studied. Adolescent pregnancy is associated with maternal and neonatal mortality,[3–6] preterm delivery,[3–5,7–11] low birth weight,[3–5,7–10,12,13] and infants being born small for gestational age.[5,8,9]

Less is known about the impact of early pregnancy on the growth of the young mother herself. If pregnancy occurs while a young woman is still growing, it could lead to competition between mother and fetus for nutrients, potentially reducing attained height in comparison to that which would have been achieved in the absence of pregnancy.[14,15] Longitudinal studies have demonstrated that there is potential for an appreciable degree of linear growth among girls following menarche.[16–19] In the Fels Longitudinal Study, the median increase in stature following menarche was 7.4 cm, and the median age of attainment of adult stature among girls was 17.3 years.[16–18] Frisch found that from menarche to 18 years, girls grew an average of 7.1 cm.[19] In the Camden Adolescent Pregnancy and Nutrition Project, over 55% of adolescents increased in height following pregnancy.[20]

There are few studies that have investigated the association between adolescent pregnancy and adult attained height, and the findings are conflicting. Studies of populations in Brazil, India, and the U.S. have reported a significant reduction, ranging from 0.3 to 3.0 cm, in attained height following adolescent pregnancy,[21–23] however, two of the studies only found an effect in select groups within their study populations. Another study of a U.S. population found no difference in attained height between girls experiencing an adolescent pregnancy and girls who did not.[24] Several of these prior studies controlled for age at menarche, however, in general, they did not control for other proximate predictors of adolescent pregnancy risk, such height immediately preceding the adolescent pregnancy.

In this analysis, we investigated the association between adolescent pregnancy and attained height among black girls in Soweto, South Africa. To our knowledge, ours is the first study to examine this association in a sub-Saharan African population with a high prevalence of adolescent pregnancy. Unlike many prior studies on this topic, we relied on a prospective design and accounted for characteristics that are related to both the risk of adolescent pregnancy and adult attained height.

## Methods

We analyzed data from the Birth to Twenty Plus study, a birth cohort that began in 1990 in Soweto, the urban township adjacent to Johannesburg in South Africa. Detailed information on this cohort has been published elsewhere.[25] The study enrolled pregnant women at gestational age 26–40 weeks, who were expected to deliver during a 6-week period in early 1990. Participants were predominantly Black women with low socioeconomic status. The study was designed to track prospectively the growth, health, well-being and educational progress of their children. Throughout the study, participants or their caregivers provided written informed consent, and ethical approval was obtained from the University of the Witwatersrand Committee for Research on Human Subjects (approval ID #M010556).

The children’s serial height data were used for this analysis. Weight at birth and weight and length (or height after age 2 y) were measured on up to 17 occasions through age 20 y using standard procedures.[26] Supine length (<24 mo) and standing height (≥24 mo) measurements were converted to height-for-age z scores (HAZ) using the WHO references.[27–29] We removed from the analysis heights/HAZ scores where the HAZ was implausible, defined as |HAZ| > 5 at any one round or an absolute value of change in HAZ between rounds > 4. Reproductive histories were updated at each study visit.

We restricted our study sample to Black female offspring (n = 1,329), as this group comprised 80% of the offspring cohort and there were too few adolescent pregnancies among girls of other ethnicities to permit meaningful analysis. To be eligible for matching and analysis, a girl had to have birth weight (birth length was not measured), an attained height (considered to be age 17 y or later) and at least one intermediate measure of height. Attained height (cm) was defined based on the oldest age at which a height measurement was available (20 y, 18 y, or 17 y). After these inclusion criteria, there remained 840 eligible females. Of the 840 eligible girls, 59 (7%) reported conception prior to age 17 y. Of those, five experienced miscarriage or voluntary abortion of the pregnancy, the precise dates of which were not available. No girls reported two pregnancies before age 17 y. We matched each girl who conceived before age 17 y and went on to give birth (hereafter denoted as the early pregnancy group, n = 54) to one girl who did not become pregnant prior to age 20y (comparison group, n = 54), based on age at self-reported menarche (in integer years) and on HAZ from the year before the early pregnancy occurred (all pairs were within 0.14 SD). The onset of menarche, in addition to being a prerequisite for pregnancy, is an indicator of pubertal development, the timing of which impacts adult height in girls.[30,31] Matching on age was not necessary, as the girls were all born within fourteen weeks of each other. The final analytical sample included 54 matched pairs. The remaining 732 females (none of whom had become pregnant by age 17 y) were considered as an internal reference group.

### Statistical Methods

Descriptive statistics were used to present the distribution of key demographic, socioeconomic, and anthropometric characteristics. The socioeconomic variable ‘assets’ is a measure of the wealth of the child’s caretakers at the time of the girl’s birth (1990), reported in quintiles (1-poorest; 5-wealthiest), based on home type, home ownership, electricity in the home, and ownership of a car, refrigerator, washing machine, or phone. The income variable is a measure of the annual income in 1990 (in Rands) of those supporting the child.

We compared characteristics between the early pregnancy and comparison groups using paired t-tests, the Wilcoxon signed rank test, McNemar’s chi-square test for paired proportions, and the Sign test. We also compared characteristics between the early pregnancy group and all other girls in the cohort (excluding the comparison group; n = 732) using the two-sample t-test, the Wilcoxon rank sum test, and Fisher’s exact test. Mean height and HAZ at each measurement from three months to 18 years were plotted. We used the paired t-test to determine whether there was a statistically significant difference in adult height between the early-pregnancy and the comparison groups. We calculated the within-pair difference in adult height, and used this difference as the outcome in a linear regression model, with adjustment for the within-pair differences in maternal height and pre-pregnancy HAZ and an indicator variable representing whether there was a subsequent second pregnancy before the adult height measurement was taken (which occurred for 5 girls; all these pregnancies were at ages >17 y). As maternal height was only available for both members of 26 matched pairs, we ran the regression models with and without this variable. The Intercooled STATA 10.0 (StataCorp, College Station, Texas) statistical program was used for all data analyses.

## Results

The mean age at conception for the early pregnancy group was 15.9 years (range: 13.7 to 16.9 y). There were no statistically significant differences between the early pregnancy and comparison groups for several demographic and socioeconomic factors (Table 1). Compared to all other girls, girls in the early pregnancy group had mothers with significantly lower mean years of schooling (p<0.01), had a lower age at menarche (p<0.001), and were taller at 11y (p = 0.04) and 12y (p<0.01).

**Table 1:** Selected Demographic, Socioeconomic, and Anthropometric Characteristics of the Early Pregnancy Group, the Comparison Group and All other Females in the Birth-to-Twenty Plus Cohort.

	Early Pregnancy^1^	Matched Comparison^2^	All other females in the cohort^3^
	(n = 54)	(n = 54)	(n = 732)
Maternal height, cm	158.7 ± 6.1	159.5 ± 6.4	158.5 ± 6.0
Maternal schooling, y	8.3 ± 3.2	9.7 ± 2.4^a^	9.7 ± 2.7^b^
Maternal parity	2.4 ± 1.7	2.3 ± 1.3	2.1 ± 1.3
Maternal annual income, Rands	9900 ± 9200	11300 ± 8700	9400 ± 7800
Maternal assets/wealth quintiles, %			
1 (lowest)	18.8	18.8	15.8
2	18.8	18.8	18.1
3	33.3	22.9	36
4	20.8	20.8	19.2
5 (highest)	8.3	18.8	10.9
Age at menarche, y	12.2 ± 1.0	12.2 ± 1.0	12.8 ± 1.2^b^
Age at conception, y	15.9 ± 0.8	-	-
Height at ages prior to matching, cm			
11y	149.0 ± 5.4	148.8 ± 6.3	145.9 ± 7.5^b^
12y	155.0 ± 5.6	154.1 ± 6.1	151.3 ± 6.8^b^
Height at matching, cm	159.4 ± 5.5	159.3 ± 5.5	-
HAZ at matching	-0.3 ± 0.8	-0.3 ± 0.8	-
Attained height, cm	160.4 ± 5.5	160.3 ± 5.8	159.5 ± 6.1
Height change from matching to final height, cm^3^	0.6 (0.1, 1.7)	0.8 (0.03, 1.9)	-

Mean ± SD unless otherwise specified; tests of statistical significance performed on early pregnancy vs. comparison groups, as well as the early pregnancy group vs. all other girls in the cohort.

^1^ Conception prior to age 17.

^2^ No conception prior to age 20 y, matched on age at menarche and height immediately before early-pregnancy group conception.

^3^ Attained height—height at matching.

^a^ Significant difference between early pregnancy and comparison group (p<0.05).

^b^ Significant difference between early pregnancy group and all other girls in the cohort (p<0.05).

**Fig 1:**
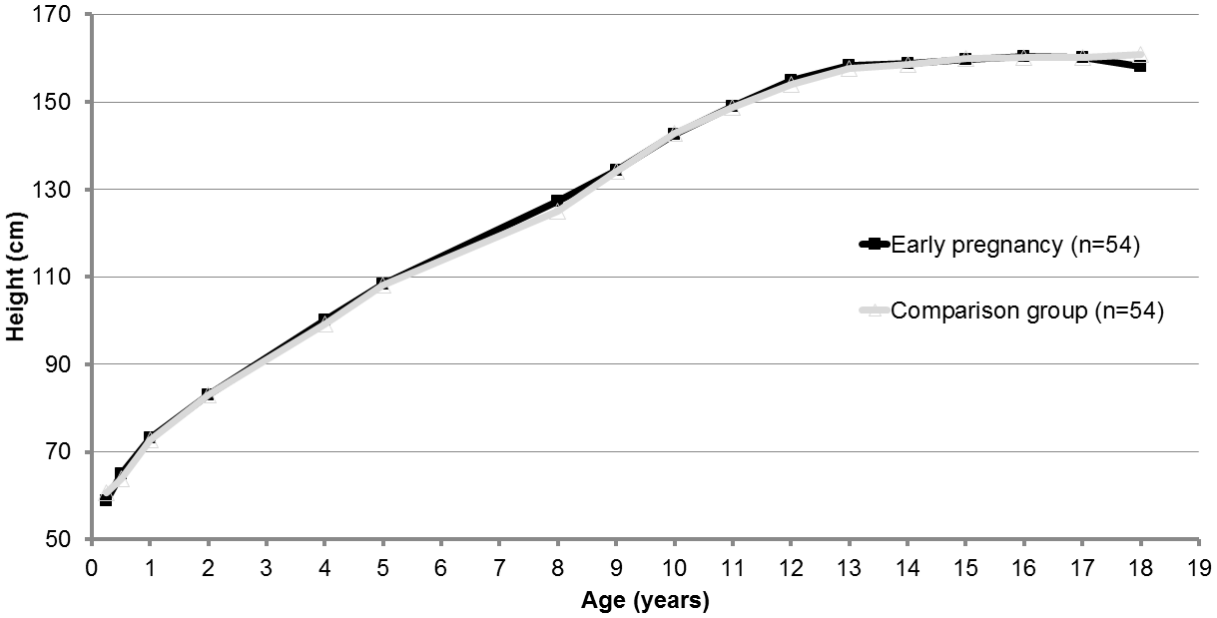
Mean height (cm) by age, matched-paired girls in the Birth-to-Twenty Plus Study.

**Fig 2:**
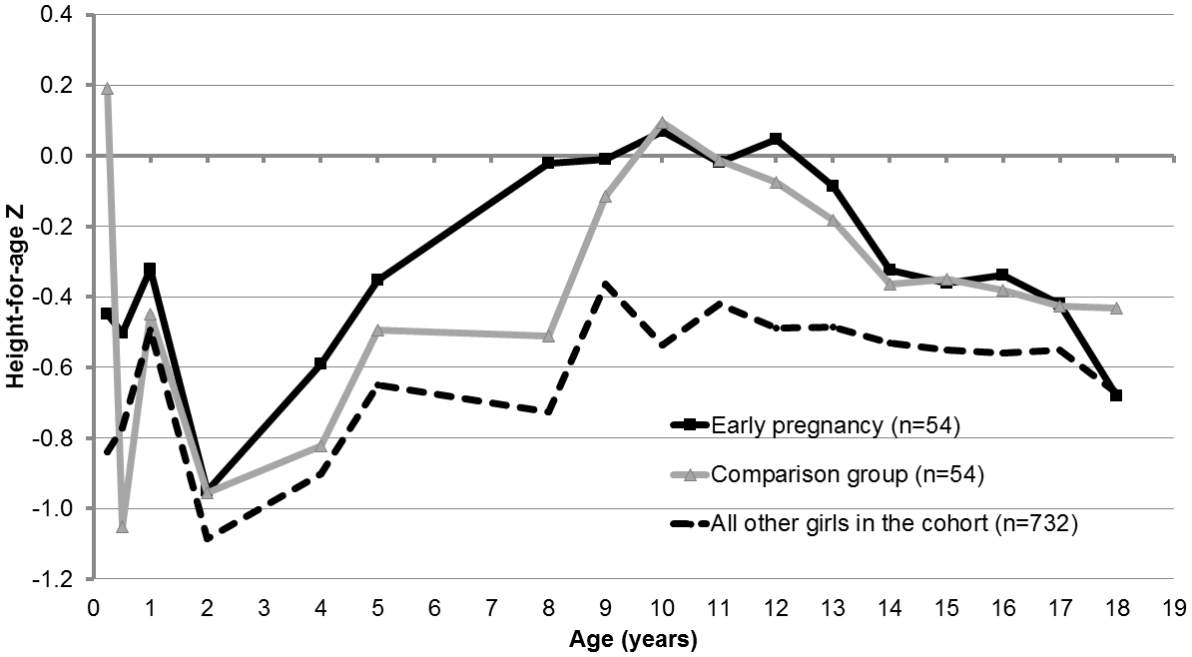
Mean Height-for-age Z score by age, girls in the Birth-to-Twenty Plus study.

For both the early pregnancy and comparison groups, adult height was reached after 15 years of age, and the growth trajectories for both groups were nearly identical (Fig 1). Compared to all other girls in the cohort, the early-pregnancy and comparison groups had a higher HAZ; this difference was especially pronounced in the pre- and peri-pubescent years (Fig 2). The median change in height from matching to adulthood was 0.6 cm for the early pregnancy group and 0.8 cm for the comparison group (p = 0.9). The mean adult height, on average at 18.5 y, was 160.4 cm for the early pregnancy group, versus 160.3 cm for the comparison group (p = 0.7).

In the linear regression model, none of the within-pair differences in maternal height (p = 0.5), pre-pregnancy matched HAZ scores (p = 0.4), or whether the girl in early-pregnancy group experienced a second pregnancy (p = 0.7) were significantly related to within-pair difference in attained height (Table 2). In the larger sample available when within-pair difference in maternal height was removed from the model, these inferences were unchanged.

**Table 2:** Multiple regression analysis of within-pair differences in attained height among 54 women who conceived prior to age 17 y and matched pairs, Birth to Twenty Cohort.

**Model 1**^1^			
Intercept	0.1	0.4	(-0.6, 0.9)
Maternal height difference	0	0.1	(-0.1, 0.2)
Matching HAZ difference	-12.9	13.9	(-41.8, 15.9)
Second pregnancy—case	-0.5	1.3	(-3.1, 2.2)
**Model 2**^2^			
Intercept	0	0.3	(-0.6, 0.5)
Matching HAZ difference	14.7	8.6	(-2.6, 32.0)
Second pregnancy—case	0.6	0.9	(-1.2, 2.3)

^1^ Within-pair difference in maternal height, difference in matching HAZ, and a subsequent second pregnancy in the case before the adult height measurement was taken; n = 26 pairs because only 26 pairs had maternal height information.

^2^ Difference in matching HAZ and a subsequent second pregnancy in the case before the adult height measurement was taken; n = 54 pairs.

## Discussion

The adverse effects of adolescent pregnancy on birth outcomes, such as maternal and neonatal mortality,[3–6] preterm delivery,[3–5,7–11] low birth weight,[3–5,7–10,12,13] and small for gestational age,[5,8,9] have been well established. Teenage pregnancy has been associated with higher rates of school drop-out for young mothers and higher risk for living below the poverty line.[32,33] However, far less is known about the impact of teenage pregnancy on the growth of the young mother. We used data from the prospective Birth to Twenty Plus cohort study in Soweto, South Africa to examine the association between pregnancy before age 17 y and the attained height of the young mother. We found no difference in final adult height between girls who experienced an early pregnancy and those who did not, after controlling for key factors that might otherwise confound this association.

Several studies have examined growth during and immediately following adolescent pregnancy to determine its short-term effect on the growth of the young mother. In the Camden Adolescent Pregnancy and Nutrition Project (New Jersey, USA), 56.5% of pregnant adolescents (average age 15.6 y) made gains in height during pregnancy, as determined by knee height measurements.[20] However, other studies found that adolescent pregnancy halted short-term growth. In Mexico, Casanueva et al. matched non-pregnant adolescents with girls who had a pregnancy at ≤ 17 years (average 15.3 y), based on socioeconomic status, chronological age, age at menarche, and body mass index, and found that pregnant adolescents did not make gains in height during the 5-month follow-up period, whereas the non-pregnant controls grew 0.94 cm on average.[34] They concluded that pregnant adolescents appear to adjust their resting energy needs by ceasing growth.[34] Similarly, in Bangladesh, when adolescent primigravidae girls (average age 16.3 y) were matched with non-pregnant controls based on age and time since menarche, it was found that from the first trimester to six months postpartum, pregnant girls did not gain in stature, compared with the non-pregnant girls who grew 0.35 cm on average.[35]

Compared to these studies, the duration of our study was longer, with an average 3.5 year window between pre-conception and the adult height measurement. Therefore, we were able to investigate longer-run associations. Research exploring these longer-term impacts is sparse, especially in low- and middle-income countries, and the results have been inconsistent. In Pelotas, Brazil, girls who experienced more than one pregnancy between 15–18 y of age had a 1.6 cm deficit in stature relative to those who did not become pregnant (p<0.01) (after adjusting for age at menarche), but there was not a significant deficit in height among girls who had only one adolescent pregnancy.[21] In our study no girls experienced repeated pregnancies prior to age 17 y, so we cannot examine this question. In the 1998–1999 India National Family Health Survey, women reported having their first child at mean age 16.8 y, and statistically significant deficits in adult height were found among women who had one (-0.26 cm), two (-0.53 cm) or three (-0.98 cm) births before age 18 y, compared to those who delivered after age 18 y.[23] In a retrospective cohort study using U.S. National Health and Nutrition Examination Survey data from 1999 to 2004, non-Hispanic white women who had their first live birth before age 18 years were 2.97 cm shorter at adulthood (20–30 y) than non-Hispanic white women who gave birth to their first child at ≥ 18 y (p = 0.03) (after adjusting for age at menarche), with no differences in final adult height by adolescent pregnancy status among Mexican-American and non-Hispanic black women.[22] In the U.S. National Heart, Lung and Blood Growth and Health Study, Gunderson et al., adjusted for age at menarche and height at 9–10 years of age, and found no difference in adult height between those who did and did not experience an adolescent pregnancy. [24]

Our findings indicate that girls who become pregnant prior to age 17 y are taller at ages 11 and 12 than girls who did not become pregnant. This finding underscores the need to appropriately control for prior height as well as other earlier life characteristics that are related to pregnancy and attained height. Thus, prospective data such as ours are advantageous in clarifying whether it is the underlying characteristics or the pregnancy that determine attained height. Our study, which carefully matched on height prior to conception and accounted for other potential confounders, suggests that the fact of pregnancy is not, in this setting, limiting for growth.

There are several possible reasons why our study did not reveal an association between adolescent pregnancy and adult height. One explanation may be that total achievable growth occurred prior to pregnancy for many girls. In the Birth to Twenty cohort, adult height was reached at mean age 15–16 years. Girls who had reached their adult height by the time of conception are not at risk of slowed growth. Second, girls who were still growing at the time of pregnancy may have entered pregnancy sufficiently nourished to fuel both their growth and the growth of the fetus. South Africa is a middle-income country where undernutrition is not as widespread as it is in other countries in the region. Chronic undernutrition causes a delay in growth and development, allowing growth to continue during late adolescence.[36] When chronic undernutrition is combined with adolescent pregnancy, it may result in a young woman’s growth period overlapping with her pregnancy, thus creating competition between mother and fetus for nutrients to fuel growth.[14] From a public health perspective, there is value in focusing attention on delaying pregnancy until adult height is reached, and these efforts may be particularly important in countries where nutritional status is poor, and thus skeletal maturation may be delayed.

This study makes a methodological contribution to the literature on adolescent pregnancy and adult height. The use of data from a prospective birth cohort allowed us to match on factors collected prior to conception, and to carefully control for proximate predictors of adolescent pregnancy risk. Several of the previous studies controlled for age at menarche, however, to our knowledge, this is the only study to control for physical maturity (height) immediately preceding the adolescent pregnancy. This is important because physical maturity is predictive of early puberty [37], which then increases the risk of early onset of sexual activity. We found that girls who had an early pregnancy experienced menarche onset at an earlier age and were taller at 11y and 12y of age than other girls in the cohort. These factors increase risk of adolescent pregnancy and impact adult height; however, by controlling for them through our matched-pair design, we found no association between adolescent pregnancy and adult height. A final limitation of our study was the relative infrequency of adolescent pregnancy in this population, resulting in a small number of matched pairs for analysis.

## Conclusion

We found that among black girls living in Soweto, South Africa, pregnancy before age 17 y was not associated with adult attained height.
